# Novel insights into the isolation of extracellular vesicles by anion exchange chromatography

**DOI:** 10.3389/fbioe.2023.1298892

**Published:** 2024-01-19

**Authors:** Leon F. Koch, Tatjana Best, Elena Wüstenhagen, Klaus Adrian, Oliver Rammo, Meike J. Saul

**Affiliations:** ^1^ Department of Biology, Technische Universität Darmstadt, Darmstadt, Germany; ^2^ Merck Life Science KGaA, Darmstadt, Germany; ^3^ Department of Oncology, Hematology and Bone Marrow Transplantation with Section Pneumology, Universtiy Cancer Center Hamburg, University Clinic Hamburg-Eppendorf, Hamburg, Germany

**Keywords:** extracellular vesicles, ion-exchange chromatography, isolation, Eshmuno^®^ Q, downstream processing, scalability, charge-based

## Abstract

Extracellular vesicles (EVs) are membrane structures enclosed by a lipid bilayer that are released into the extracellular space by all types of cells. EVs are involved in many physiological processes by transporting biologically active substances. Interest in EVs for diagnostic biomarker research and therapeutic drug delivery applications has increased in recent years. The realization of the full therapeutic potential of EVs is currently hampered by the lack of a suitable technology for the isolation and purification of EVs for downstream pharmaceutical applications. Anion Exchange Chromatography (AEX) is an established method in which specific charges on the AEX matrix can exploit charges on the surface of EVs and their interactions to provide a productive and scalable separation and purification method. The established AEX method using Eshmuno^®^ Q, a strong tentacle anion exchange resin, was used to demonstrate the principal feasibility of AEX-based isolation and gain insight into isolated EV properties. Using several EV analysis techniques to provide a more detailed insight into EV populations during AEX isolation, we demonstrated that although the composition of CD9/63/81 remained constant for tetraspanin positive EVs, the size distribution and purity changed during elution. Higher salt concentrations eluted larger tetraspanin negative vesicles.

## 1 Introduction

Extracellular vesicles (EVs) are membrane-bound particles (50–200 nm in diameter) released by almost all cell types. In recent years, EVs have been shown to play an important role in cellular communication (Raposo and Stoorvogel, 2013; van Niel et al., 2018). As part of their functional cargo, EVs can transfer bioactive proteins, lipids and nucleic acids from donor to recipient cells. In doing so, they regulate various signaling processes (Mathieu et al., 2019; Yates et al., 2022).

Additionally, components of EVs can also be used as diagnostic targets for liquid biopsies, providing an indication of the characteristics of the donor cell (Liu et al., 2022). Furthermore, EVs can naturally carry therapeutic agents or be modified by physical, chemical or bioengineering strategies (Jafari et al., 2020). Because of their excellent biocompatibility and stability, EVs are ideal nanocarriers for bioactive ingredients that can target specific cell types for signaling, immunoregulatory or other therapeutic effects (Herrmann et al., 2021; Song et al., 2022; Zheng et al., 2022). For example, EVs secreted by mesenchymal stem cells (MSCs) are widely used in regenerative medicine. Their cargos promote tissue repair and have anti-inflammatory effects (Kamerkar et al., 2017). Immune cell-derived EVs, which can confer unique immunomodulatory properties to recipient tumor cells, are widely used in cancer therapy (Li et al., 2022). And finally, EV-based vaccines typically make use of EVs that carry, or can be modified to carry, both antigens and adjuvants (Santos and Almeida, 2021).

Since different EVs may be suitable for different therapeutic applications, the choice of EV type is a critical factor for any EV application (Wiklander et al., 2019). Upscaling cell culture conditions and purification methods under standardized conditions is required to transfer EV therapeutics into clinical trials and industrial scale (Klyachko et al., 2020). EV production is at an early stage of development for upscaling to industrial scale. The cell culture methods will have to be individually adapted to the specific cell types and to the required quantities of EVs. Two recent studies have described the production of EVs from MSCs and cardiac progenitor cells (CPCs) in clinically relevant amounts, both in a bioreactor and in a HyperStack™ system (Andriolo et al., 2018; Mendt et al., 2018). No phenotypic changes were observed in either the cells or the EVs when the cell culture process was scaled up (Andriolo et al., 2018; Mendt et al., 2018). The process of isolating EVs on a large scale is another hurdle for the therapeutic application of EVs (Klyachko et al., 2020). This process must consider the required purity of the preparation and has to ensure the functionality of the vesicles. Several EV isolation methods are currently being developed and used. However, most of these methods have one or more drawbacks that result in either insufficient purity, limited scalability, combined with potentially high manufacturing costs (Paganini et al., 2019). Typically, tangential flow fractionation (TFF) is used as the first diafiltration and concentration step in an EV downstream process (Andriolo et al., 2018). After TFF, further purification and separation of EVs is often performed by ultracentrifugation (UC), polyethylene glycol (PEG)-based precipitation, affinity-based chromatography, or size exclusion chromatography (SEC) (Corso et al., 2017; Andriolo et al., 2018; Ludwig et al., 2018; Staubach et al., 2021). UC is difficult to scale up and problematic to scale out. Size exclusion chromatography is scalable to a limited extent, but does not reduce the high volume of EV-containing feed (Stulík et al., 2003).

In order to overcome these problems, new techniques or already known methods, such as ion exchange chromatography (IEX), need to be developed and further adapted to the scalable EV isolation process. IEX has been successfully used in the clinical production of several monoclonal antibodies (Hardin et al., 2009) and is considered a key element in the robust and cost-effective production of future drugs (Bhattacharyya, 2012; Lu and Qi, 2021; Yuan et al., 2023). For the purification of EV, we chose to use tentacle ion exchangers, which are commonly used chromatography media with grafted polymers. Graft polymer tentacle surfaces are available with different functionalities such as anion or cation exchange groups that expand into the porous bead network to interact with biomolecule surface charge groups at low salt concentrations (Pee et al., 2016). In bind-elute mode, high recoveries, small elution volumes and less peak broadening are achieved by switching to a high salt concentration for efficient release of the target molecule (Thomas et al., 2013). Due to their highly flexible network, tentacle ligands also allow for multi-point interactions with the target molecule. This makes them attractive for the removal or capture of larger molecules such as viruses and enveloped virus-like particles (Pee et al., 2016; Pereira Aguilar et al., 2019). Because their physical and chemical properties are comparable to those of many cellular EVs (Noltet Hoen et al., 2016), we used tentacle polymers for IEX-based purification in this study. We have shown that this technology is ideal for EV purification. A particular focus was to better understand the behavior of EVs during the purification process. For the first time, we combined different methods to analyze EVs and comprehensively characterized how and which populations of EVs are purified by IEX.

## 2 Materials and methods

### 2.1 Cell culture

To obtain EV containing conditioned cell onditioned media, suspension-adapted human embryonic kidney (HEK293) cells (VP002, SAFC, Sigma-Aldrich, St. Louis, United States) were cultivated in EX-CELL*®* CD HEK293 Viral Vector medium (14385C, SAFC, Sigma-Aldrich, St. Louis, United States) supplemented with 6 mM L-glutamine (59202C, SAFC, Sigma-Aldrich, St. Louis, United States) according to manufacturer’s instructions.

For larger EV production, cells were scaled-up consecutively and seeded into a Mobius*®* 3L single-use bioreactor (CR0003L200, Millipore, Burlington, MA, United States) at a cell density of 1 × 10^6^ viable cells per mL (VC/mL). EV-containing conditioned cell conditioned media (CM) was harvested after 48 h of cultivation at a cell density of 4 × 10^6^ VC/mL and cell viability of around 95%. CM was then centrifuged at 2000 x g for 20 min at 4°C, aliquoted in 200 mL fractions and stored at −80°C until further use.

For the generation of Jurkat conditioned media, cells were seeded in RPMI medium (Sigma-Aldrich, St. Louis, United States) supplemented with 5% insulin-transferrin-selenium solution (Sigma-Aldrich, St. Louis, United States). After incubation the CM was centrifuged for 20 min at 2000 x g, 4°C, filtered using 0.22 µm syringe filter and stored at −80°C until further use.

### 2.2 Pretreatment of cell culture supernatant

200 mL of frozen CM was thawed in a water bath and warmed to 37°C. DNAwas digested with 100 U/mL of Benzonase® (Millipore, Burlington, United States) for 1 h, supplemented with a final MgCl_2_ concentration of 2 μM at 37°C. The CM was then clarified using a 0.22 µm syringe filter (Millex-GP, Millipore, Burlington, United States) and concentrated using a Centricon® Plus-70 centrifugal filter (Millipore, Burlington, United States) with a 30 kDa cut-off. The medium was concentrated at 3,500 x g for 15–20 min and replenished until it was reduced to 10.5 mL. 10 mL of the concentrated medium was used for EV isolation and the remaining 0.5 mL were used for analysis.

### 2.3 EV isolation by chromatography

EVs were isolated on an Äkta Pure™ 25 M (Cytiva, Marlborough, United States) equipped with a fraction collector (F9-C, Cytiva, Marlborough, United States) using a 1 mL Eshmuno^®^ Q column (MiniChrom, Merck, Darmstadt, Gemany). All reagents used during chromatography were filtered through a 0.1 µm membrane filter (VacuCap™ 90, Pall Life Sciences). Steps were performed at room temperature and the column was equilibrated with wash buffer to remove residual storage buffer (150 mM NaCl, 20% EtOH). Chromatography was performed at 1 mL/min except for sample application (0.5 mL/min). For EV isolation the column was equilibrated for 10 column volume (CV), then concentrated CM was applied until air was detected and the injection was terminated with 1.5 mL wash buffer. The column was washed for 10 CV followed by gradient elution starting with 0% buffer B (2 M NaCl) leading to 60% B over 15 CV. After elution, the column was stripped with 100% B over 10 CV and a cleaning in place (CIP) was performed with 0.5 M NaOH for 10 CV. The column was re-equilibrated with wash buffer and stored in storage buffer. Flow-through (FT), washing (W), stripping (S) and CIP were collected in 2 mL fractions (96-deepwell plate, Protein LoBind^®^, Eppendorf, Hamburg, Germany) while the elution was fractionated in 0.5 mL steps. Once the chromatography was complete, the fractions were aliquoted and stored either at 4°C (short term; up to 4 days) or −80°C for subsequent analysis. Buffer compositions and detailed chromatography steps are given in Supplementary Table S1, S2. Chromatography steps were monitored at 260 and 280 nm and by an inline multiangle light scattering (MALS) detector. Particles were tracked and analyzed during chromatography using a Dawn^®^ 8 MALS detector (Wyatt Technology, United States) operating at 10% laser power and 3 reads/sec. Peaks were set manually and particles were analyzed using the Astra software Version 8.1.1.12.

Unspecific EV adsorption study was performed by isolating EVs from concentrated HEK293 CM and pooling elution fraction. Pooled elution fractions were then reapplied to the same Äkta™ system without column, after it has been cleaned with 0.5 M NaOH. Sample application was monitored using UV detection and MALS and all particle containing fractions were analyzed. Salt stability was assessed by pooling EV containing elution fractions, performing a buffer exchange thrice with 125 mM NaCl buffer, using a 10 kDa cut-off ultracentrifugation device (Amicon^®^ ultra.15, Millipore, Burlington, MA, United States). Afterwards, EVs were diluted with appropriate salt buffers to achieve a final NaCl concentration of: 125 mM, 250 mM, 500 mM, 1000 mM and 1500 mM. EVs were stored at 4°C and measured immediately, after 1 day and after 4 days.

### 2.4 EV characterization

#### 2.4.1 Nanoparticle tracking analysis (NTA)

Size distribution and particle concentration of EV samples were analyzed on a NanoSight NS300 (Malvern Panalytical, Malvern, United Kingdom), equipped with a 532 nm laser module and NanoSight Sample Assistant (Malvern Panalytical, Malvern, United Kingdom). Particle tracking and subsequent calculations were performed using NanoSight Software Version 3.4. For EV measurements, 5 videos of 60 s were acquired with the manual setting focused on camera level 16, at a speed of 30 at 25°C and analyzed at threshold level 5. Samples were diluted in 0.1 µm filtered washing buffer (125 mM NaCl, 50 mM Tris, pH 7.4) to achieve particles/frames of 20–35 with the aim to reduce concentration dependent changes in particle quantification.

#### 2.4.2 Bicinchoninic acid assay (BCA)

For the determination of protein concentration, EV samples were analyzed in a 96-well plate (Greiner Bio-One GmbH, Kremsmünster, Germany) in two technical replicates using QuantiPro™ BCA Assay Kit (Sigma-Aldrich, St. Louis, United States) according to the manufacture’s instructions. Bovine serum albumin (BSA) was used for the standard curve, assay was incubated in the dark at RT for 16 h and the absorbance was measured at 562 nm in an Infinite M Nano Microplate Reader (Tecan, Maennedorf, Switzerland).

#### 2.4.3 Single particle interferometric reflectance imaging sensor (SP-IRIS) with immunofluorescence staining

Antibody coated microarray chips (Unchained labs, Pleasanton, United States) against CD9 (clone HI9a), CD63 (clone H5C6) and CD81 (clone JS-81) were pre-scanned to establish baseline of adherent particles prior to sample incubation. Samples were diluted appropriately in incubation solution (Solution I, Unchained labs, Pleasanton, United States) to avoid oversaturation and 50 µL were incubated on the chips overnight in an airtight 24-well plate at room temperature. The next day, staining was performed according to the manufacturer’s instructions. Briefly, the chips were washed 3 times in Solution A (Unchained labs, Pleasanton, United States) and then incubated for 1 h in the antibody detection mixture (containing anti-CD9 CF^®^488; anti-CD63 CF^®^647; anti-CD81 CF^®^555). The chips were then washed once with Solution A and 3 times with Solution B (Unchained labs, Pleasanton, United States). Finally, the chips were washed once with Milli-Q purified (MQ) H_2_O and dried by removing the chips from the solution at a 45° angle. The chips were allowed to dry completely. They were then imaged on an ExoView™ R100 (Unchained labs, Pleasanton, United States) and analyzed using the ExoView™ software version 3.1.4.

#### 2.4.4 Western blotting

For Western blot analysis, cells were lysed with Radioimmunoprecipitation assay (RIPA, 50 mM Tris; 150 mM NaCl; 1% NP-40; 0.1% sodium dodecyl sulfate (SDS); 0.1% deoxycholic acid) buffer containing protease inhibitor cocktail (cOmplete Mini, EDTA-free, Roche, Basel, Switzerland) for 15 min on ice. Protein concentration was measured by BCA. A constant volume of 30 µL was loaded for EV samples and a fixed protein amount of 50 µg was loaded for cell lysates. All samples were diluted with 4x loading dye (200 mM Tris; 8% SDS; 6 mM Bromphenol blue, 4,3 M Glycerol) and boiled at 95°C for 5 min. Proteins were separated on a 12% SDS gel under unreduced conditions, run at 130 V and then blotted onto nitrocellulose membrane (LI-COR Biosciences, Lincoln, United States). Membranes were blocked for 1 h in blocking buffer (EveryBlot, Bio-Rad, Hercules, United States) and subsequently incubated with antibodies against CD9 (Clone ALB6, Santa Cruz Biotechnology, Dallas, US), CD63 (Clone H5C6, Novus Biologicals, Littleton, US), CD81 (Clone 5A6, Merck, Darmstadt, Germany), Histone-3 (Clone 1B1-B2, Biolegend, San Diego, US), Calnexin (C4731, Sigma-Aldrich, St. Louis, US) and Syntenin-1 (Clone C-3, Santa Cruz Biotechnology, Dallas, US) for either 2 h at RT or at overnight at 4°C. Blots were washed three times with PBST (137 mM NaCl; 2.7 mM KCl; 10 mM Na_2_HPO_4_; 1.8 mM KH_2_PO_4_; 0.1% Tween20) and incubated with appropriate infrared dye-conjugated secondary antibodies for 45 min at RT. The blots were washed three times with PBST prior to detection of protein bands on the Odyssey FC reader (LI-COR Biosciences, Lincoln, United States).

#### 2.4.5 Enzyme-linked immunosorbent assay (ELISA)

Intact CD81positive EVs were analyzed using the CD81-Capture Human Exosome ELISA Kit (FUJIFILM Wako Chemicals Europe, Neuss, Germany) according to the manufacturer’s instructions. Briefly, samples were diluted appropriately in sample reaction buffer and incubated for 2 h on prewashed antibody-immobilized plates. The plate was washed and then incubated for 1 h with biotinylated antibody (targeting CD81). The plate was again washed and incubated for 2 h with HRP-conjugated streptavidin. The solution was discarded, and the plate was washed. 3.3′,5.5′-Tetramethylbenzidin (TMB) substrate was added for 30 min and terminated using stop solution. The plate was shaken for 5 min and then absorbance was measured at 450 nm with 620 nm as reference using a microplate reader (Tecan Infinite M Nano, Tecan, Maennedorf, Switzerland).

#### 2.4.6 Transmission electron microscopy (TEM)

Samples were incubated for 10 min on glow discharged, copper grids coated with carbon-formvar (Plano GmbH, Wetzlar, Germany) and fixed in 2% formaldehyde (Car Roth, Karlsruhe, Germany) for 10 min at RT. Excess liquid was blotted off and the grids were washed twice with MQ H_2_0 and dried completely. Prior to imaging, the grids were immersed in 4% uranyl acetate for 1 min, then washed twice in MQ H_2_0 and dried again. Images were acquired on a Zeiss EM109 electron microscope (Carl Zeiss, Oberkochen, Germany) operating at 40 kV and equipped with a BioScan Camera Model 792 (Gatan Inc., Pleasanton, United States).

#### 2.4.7 Asymmetric-flow field-flow fractionation (AF4)

AF4 was performed on an Eclipse^®^ AF4 system (Wyatt Technology Europe, Dernbach, Germany) coupled with an Agilent HPLC 1260 system (Agilent Technologies, United States) with pump, autosampler, refractive index (RI) and UV detectors and MALS with QELS detector (Wyatt Technology Europe, Dernbach, Germany). A short channel (144 mm length and 400 µm thickness) was used with a 10 kDa molecular weight cutoff regenerated cellulose membrane for separation. The running buffer was composed of 20 mM Na_2_HPO_4_, 20 mM NaH_2_PO_4_ and 350 mM NaCl, supplemented with 250 ppm sodium azide and filtered through a 0.1 µm membrane filter. From all samples, 100 µL were injected 2 times and were run with the following method: 2 min equilibration with 2 mL/min, then the injection starts with 1 min focusing time at 2 mL/min, afterwards sample was injected in the focus mode using the injection flow of 0.2 mL/min over 7 min and then the sample was focused for 7 min at 2 mL/min. The elution was consisted of several steps, a constant elution for 5 min at starting flow 2 mL/min, then an exponential gradient to 0.05 mL/min (20 min–slope 6) and a final constant elution for 20 min at 0.05 mL/min. The fallowing 3 steps were wash steps. The channel flow was set to 1 mL/min, the detector flow to 0.5 mL/min and the above flow rates refer to the crossflow. All data were acquired and evaluated with Software Astra 8.1.1.12, using online particle and number density templates. To calculate the distribution of the molar masses, running buffer was measured as a blank and subsequently the baseline subtraction was performed.

### 2.5 Statistics and calculations

Results are presented as the mean +standard error of the mean (SEM) of either two or three independent experiments.

Statistical analysis was performed using GraphPad Prism 9 Version 9.1.2.

Chromatographic recovery is defined as followed:

Formula 1

$$Recovery=\left(\frac{Flow‐through+Wash+Elution+Strip+CIP}{Sample}\right)*100$$

## 3 Results

**FIGURE 1:**
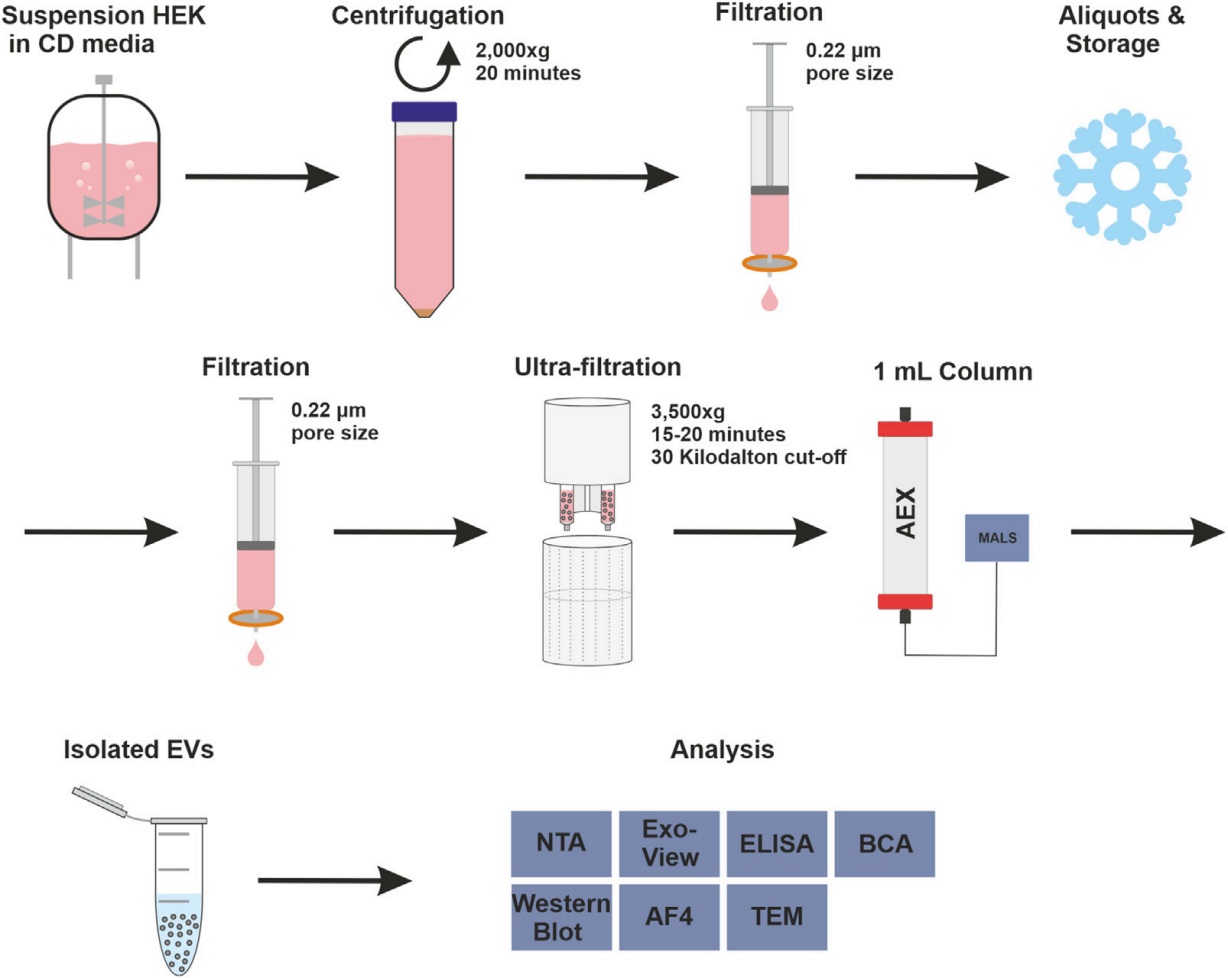
Complete isolation and purification process and overview of used analytical methods. Overview of upstream process steps with cell culture of human embryonic kidney (HEK293) cells in chemical defined (CD) media, purification with anion exchange (AEX) chromatography, coupled with multiangle light scattering (MALS), and analytical methods were used to measure isolated extracellular vesicles (EV), in detail: Nanoparticle tracking analysis (NTA), ExoView™, enzyme-linked immunosorbent assay (ELISA), bicinchoninic acid assay (BCA), Western blot, asymmetric-flow field-flow fractionation (AF4) and transmission electron microscopy (TEM).

The strong anion exchanger Eshmuno^®^ Q, a polyvinyl ether-based resin with a protruding tentacle structure for more efficient isolation (Saari et al., 2023), was selected to develop a chromatographic method for purifying EVs. The positively charged resin was chosen for its ability to specifically attract the known negative surface charges generated by phosphatidylserine, proteins and their glycosylation on the surface of EVs (Akagi et al., 2014; Deregibus et al., 2016; Woo et al., 2022). During the purification process, it is important to know how the impurities behave. Therefore, all fractions were thoroughly analyzed in a chromatographic process using various analytical methods. The process includes pretreatment steps such as filtration, nuclease treatment and concentration, and chromatographic purification of the EVs. Figure 1 shows the process and the various upstream and downstream steps as well as all analytical methods used to characterize the fractions. In the absence of a suitable isolation technique for EVs in a pharmaceutical setting, HEK293 suspension cells were chosen as a model cell line. HEK293 cells are commonly used for the production of recombinant proteins and viral vectors. In addition, several products produced by HEK293 are approved as medication (Tan et al., 2021). HEK293 cells are also frequently used for the engineering of EVs. This makes them a promising and important cell line for EV production (Komuro et al., 2022; Zhang et al., 2023).

### 3.1 Different chromatographic profiles of UV absorbance and MALS signal

**FIGURE 2:**
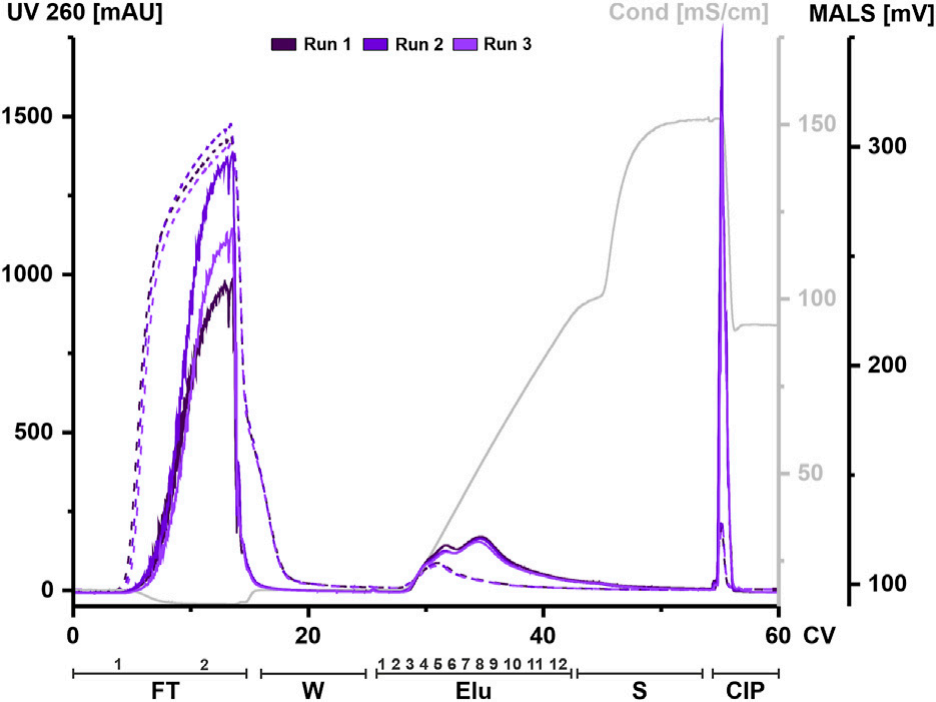
EVs from HEK293 cells can bind to and be eluted from a strong anion exchange resin. Overlay of three chromatograms of individual replicates, run 1 (dark purple), run 2 (middle purple) and run 3 (light purple) in column volume (CV) with chromatographic steps flow-through (FT), wash (W), elution (Elu), strip (S) and cleaning in place (CIP) and including fraction numbers. UV 260 (dashed line) and multiangle light scattering (MALS) traces (continuous line), as well as conductivity (light grey) are shown.

HEK293 suspension cell line was grown in a bioreactor in chemically defined medium. Cell supernatant was centrifuged to remove cell components and aliquots were frozen until further use. An aliquot was thawed at 37°C and treated with nuclease for each chromatography run. Digested CM was filtered, concentrated, and applied to a 1 mL column. The elution was carried out using a linear gradient from 125 mM up to 1.2 M NaCl. UV absorbance during chromatography at 260 and 280 nm was used to monitor nucleic acids and protein, respectively. Particles were tracked via an in-line MALS detector. All chromatographic steps were fractionated and subsequently analyzed. Figure 2 shows representative chromatograms from three independent chromatographic runs, illustrating the chromatographic steps and fractionation scheme.

Flow-through UV absorbance at 260 nm showed no difference between the three runs. However, there was a difference in MALS peak maximum between each run. The elution profile showed a peak in the UV signal at 30 CV, while the MALS signal showed two partially resolved peaks. The first peak of the MALS signal aligned with the UV signal. As the UV signal continued to decrease over time, the MALS signal showed a second peak at 35 CV until the signal begins to decrease. The UV signal returned to baseline during elution while the MALS signal shifted to strip. Both signals showed a narrow peak during CIP.

Taken together, both signals indicate that both proteins and particles such as EVs are present in the flow-through and that they co-elute during the first elution peak but not in later fractions. High signals in both the MALS and UV in the CIP indicate remaining material on the column that has not been eluted, even at high concentrations of NaCl.

### 3.2 Separation and recovery of EVs can be achieved by AEX

The CM as load for chromatography and subsequent fractions were analyzed by Nanoparticle tracking analysis (NTA) to investigate the interaction between the strong anion exchange resin and EVs. NTA is a general measurement technique that detects all light scattering particles. It is important to note that protein and EV aggregates are also detectable and therefore are indistinguishable by NTA analysis.

**FIGURE 3:**
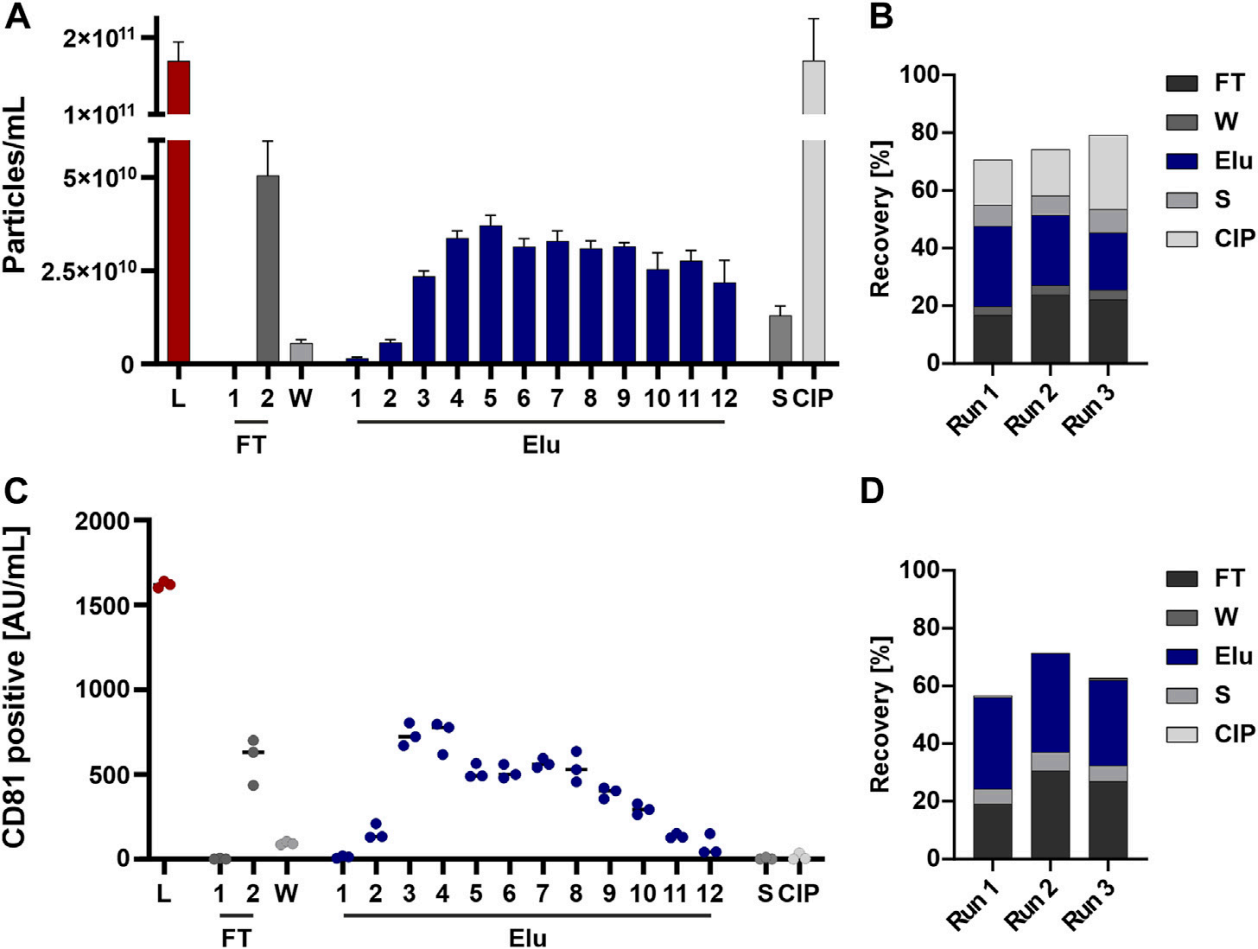
EVs from HEK293 cells can bind to and be eluted from a strong anion exchange resin. **(A)** Particle concentrations measured by nanoparticle tracking analysis (NTA) of load (L) and all chromatographic fractions flow-through (FT), wash (W), elution (Elu), strip (S) and cleaning in place (CIP) **(B)** Recovery of chromatographic steps based on NTA data. Data presented are the mean +/− SEM of three single replicates with five technical replicates. **(C)** Analysis of chromatographic fractions based on cluster of differentiation (CD) 81 presence by CD81 enzyme-linked immunosorbent assay (ELISA). **(D)** Recovery of chromatographic steps based on ELISA data. Data presented are the mean +/− SEM of three single replicates with two technical replicates.

Particles eluted throughout the elution process and were detectable in the subsequent flow-through fraction as well as strip and CIP fraction(s) (Figure 3A). The recovery based on the NTA data showed an overall recovery of 70%–80% (Figure 3B).

Generally, EVs tend to absorb to plastics, tubes, and hoses (Evtushenko et al., 2020). To analyze whether the missing EVs are lost in the system or trapped on the column, we performed a chromatographic run without the column attached to the system to see the influence of tubing and internal chromatography system structure. The NTA analysis showed a loss of 20%–30% of the EVs in the chromatography system, revealing the missing 20%–30% (Supplementary Figure S1A). EV stability in various NaCl concentration was also assessed to rule out EV loss due to instability (Supplementary Figure S1B).

The same fractions were further tested for the presence of the EV marker CD81 by ELISA to confirm the NTA data. The choice of CD81, part of the tetraspanin (TP) family which are involved in EV biogenesis (Andreu and Yáñez-Mó, 2014) and common EV marker (Breitwieser et al., 2022), for the quantification of EVs was based on the fact that this surface marker is the most highly expressed TP on the EV population of HEK293 cells. Elution fractions (Figure 3C) and EV recovery (Figure 3D) showed comparable results, whereas no CD81 signal was detected in the strip and CIP fractions.

In summary, particles were detected by NTA throughout all fractions, while CD81 positive EV showed a reduced signal in the last elution fractions and were nearly absent in Strip and CIP.

### 3.3 AEX can separate EVs on the basis of particle size

It has been extensively studied, that subpopulations of EV differ in particle size (Zhang et al., 2018; Singh et al., 2021). To further investigate if AEX can separate subpopulations of EV, we examined the particle size distribution of all fractions. Furthermore, it is well known that ultrafiltration in general can has an effect on the formation of aggregates of proteins (Arakawa et al., 2017). We therefore investigated this effect on EVs by NTA and AF4 to reveal a possible influence on the chromatographic elution behavior. AF4 is particularly useful for complex and heterogeneous samples where size separation is performed prior to size determination (Zhang et al., 2018; Zhang and Lyden, 2019). This method is also able to give information on aggregation status of samples (Liangsupree et al., 2021).

Firstly, we compared the three independent Loads using NTA but found no correlation between particle count and mean particle sizes (Sup Figure 4) that would indicate aggregate formation. However, the total particle count indicated differences when the loads, coming from the same batch of CM, were compared against each other (Sup Figure 4A).

**FIGURE 4:**
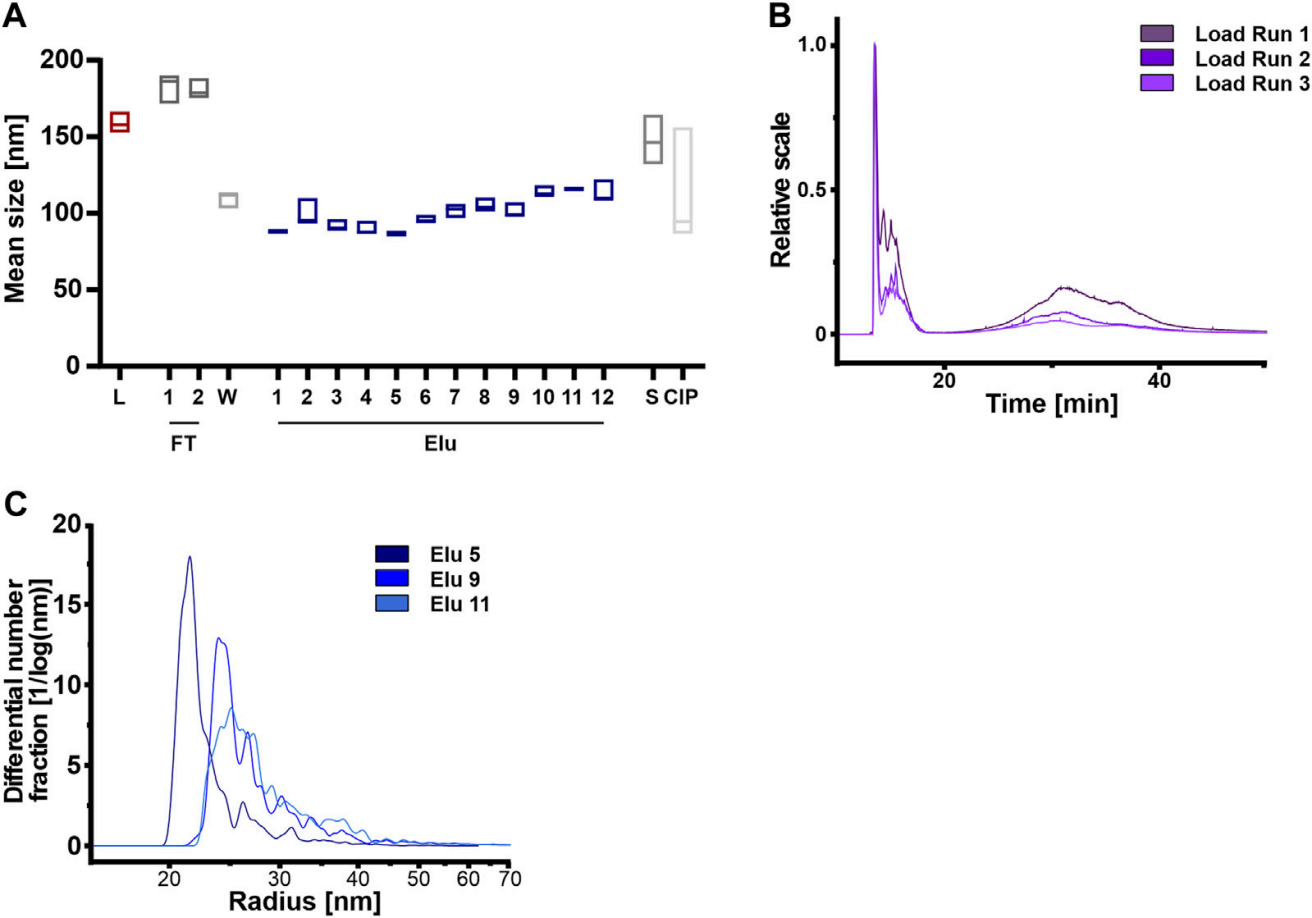
Anion exchange resin can separate HEK293 EVs on the basis of vesicle size. **(A)** Mean vesicle sizes of load (L) and chromatographic fractions flow-through (FT), wash (W), elution (Elu), strip (S) and cleaning in place (CIP), measured by nanoparticle tracking analysis (NTA). Data presented are the mean +/− SEM of three single replicates with five technical replicates. **(B)** Asymmetric-flow field-flow fractionation (AF4) chromatograms of loads from run 1 (dark purple), run 2 (middle purple) and run 3 (light purple). **(C)** Exemplary size distribution in selected elution fractions Elu 5 (dark blue), Elu 9 (middle blue) and Elu 11 (light blue) of run 1, determined with AF4. The total of all particles under the curve is 100%.

Secondly, we utilized AF4 to analyze the load in more detail (Figure 4B). Both, the 15 min and 35 min MALS curves revealed differences in particles size between the different loads, indicating a significant influence of the sample preparation before the chromatography step. At 15 min, larger particles, e.g. aggregates, eluted due to the steric elution mode (Dou et al., 2013; Zhang and Lyden, 2019). The loads of the 3 runs differed in the particle number (1.0 × 10^8^ to 3.9 × 10^9^ total particle) and size of aggregates, ranging from 130 to 270 nm. At 35 min particles with a radius of approximately 95 nm and a total particle count between 3.3 × 10^10^ and 4.7 × 10^10^ eluted.

Analyzing the elution fractions, our NTA analysis revealed that the average particle size varied significantly between samples when comparing the particle size of the load and the elution fractions (Figure 4A). Particles in the loading, flow-through and stripping fractions were larger than 150 nm. By contrast, the particles in the first elution peak were on average smaller than 100 nm, while the particles in the second elution peak had a diameter of 100–110 nm. Figure 4C displays the representative size distribution of run 1 measured by AF4. In the first peak at 30 CV (elution 5, Figure 2), 80% of elution particles were up to 32 nm in radius. Subsequent peaks (elution 9 and 11, Figure 2) displayed larger particles, where 50% of the particles had a radius of >32 nm.

In summary, the concentration variations of the particles used and the presence of aggregates possibly due to the ultrafiltration step prior to the purification step, do not affect the subsequent chromatographic purification. Larger particles and aggregates were not chromatographically bound to the column and were detected in the flow-through.

### 3.4 Changes in tetraspanin composition and colocalization on the EV envelope during AEX

To further investigate the interaction of the EV subpopulations with the column, we investigated the composition and colocalization of the TP CD9/CD63/CD81. TPs were chosen because they are enriched in EVs, are involved in their biogenesis and are generally accepted as EV markers (Jankovičová et al., 2020). Subclasses of EVs with different biochemical properties could potentially show differences in TP composition and/or colocalization.

**FIGURE 5:**
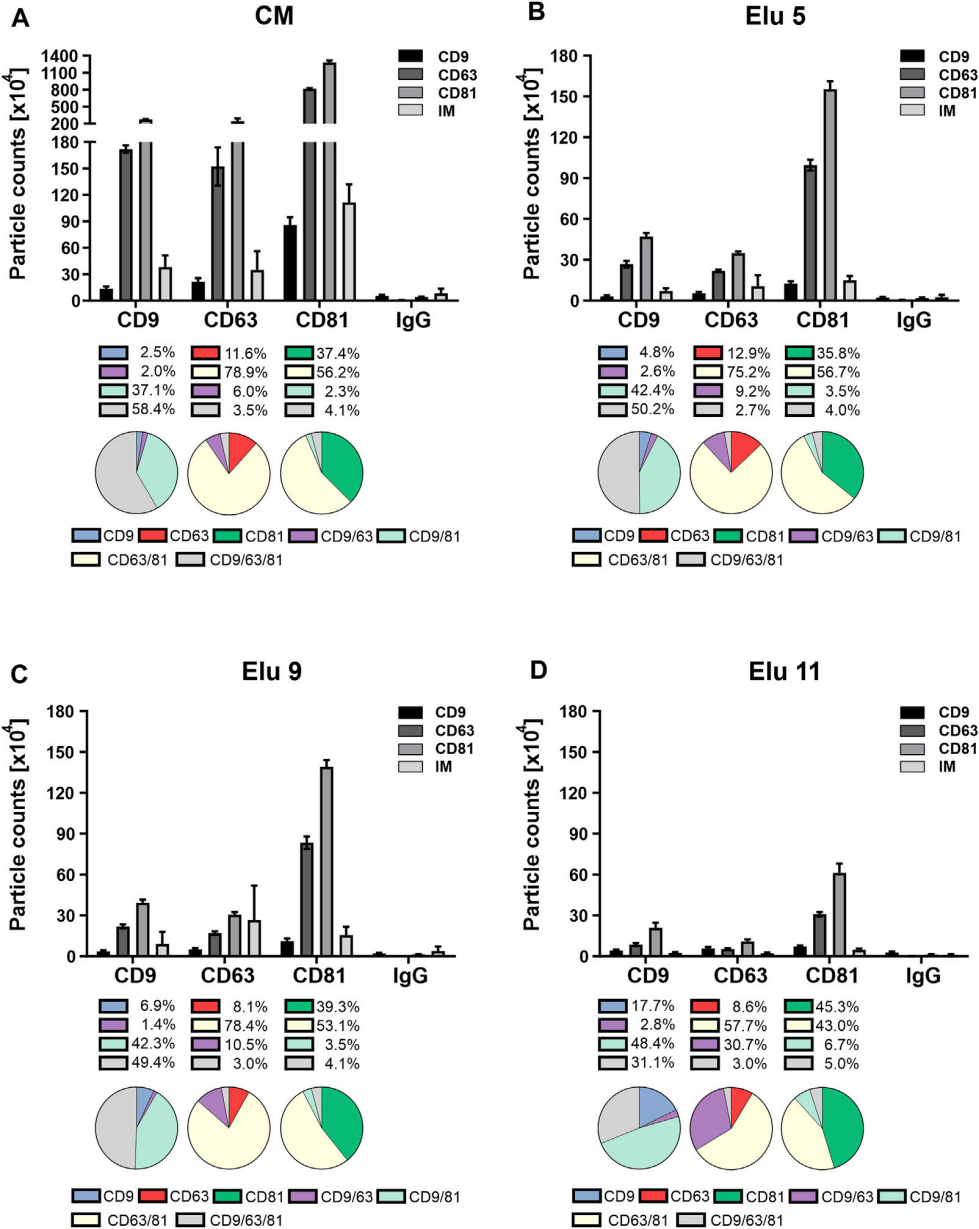
Comparison of tetraspanin (TP) composition and colocalization of concentrated conditioned media (CM) from HEK293 cells, used as load in the chromatography, and elution fractions throughout the NaCl gradient. EVs were captured using chips coated with spots against CD9, CD63, CD81 and mouse IgG as control. Fluorescent antibodies against the same TP were used for visualization and analyzed on the ExoView™ R100. **(A)** depicts TP composition and colocalization of EVs present in the load. **(B–D)** display the composition and colocalization of the elution fractions Elu 5, Elu 9 and Elu 11, respectively. Interferometric positive particles were detected using the SP-IRIS mode, with a detection threshold of 50 nm.

The ExoView™ R100 platform, a system that combines antibody-based chip capture with single particle interferometric reflectance imaging sensing (SP-IRIS), was used to observe potential changes in TP composition, colocalization and size during the isolation process. For this purpose, the load and three samples during the elution step, covering both elution peaks (elution 5 and 9) and the end of the salt gradient (elution 11), were analyzed. The pre-isolated EVs in the load (Figure 5A) showed expression of all three TP and were detected on all three capture spots. The highest number of EVs was captured on the anti-CD81 spot, followed by anti-CD63 and anti-CD9, the same profile was found on the individual capture spots. On the CD9 capture spot, more than half of the particles were positive for all three TP and one-third were double positive for CD9 and CD81. On the CD63 capture spot, the majority of EVs were double positive for CD63 and CD81. On the most capturing CD81 spot, more than half of the EVs were double positive for CD81 and CD63, followed by one-third being single positive for CD81. In addition to fluorescence detection, EVs were also detected on all three spots by interferometry (IM), with the highest number of particles on the CD81 spot.

EVs from the first elution peak (elution 5, Figure 5B) showed a very similar TP composition on all three spots with the difference that the overall particle count was lower. Colocalization on CD81 and CD63 was also identical to the load, only a slight decrease of triple positive particle was observable on the CD9 capture spot. On the CD63 capture spot, the IM positive particles doubled in comparison to the load, while the IM positive particles on the other capture spot stayed the same.

EVs from the second elution peak (elution 9, Figure 5C) showed similar particle counts compared to the first peak with an unchanged TP composition on all capture spots. The colocalization profile remained also constant compared to peak 1, with only slight changes to the load on the CD9 capture spot. IM positive particle count increased again on the CD63 capture sport and additionally on the CD9 capture spot.

Towards the end of the salt gradient (elution 11, Figure 5D), the overall particle count decreased on all spots. The TP composition on CD9 and CD81 persisted constant, while on the CD63 capture spot, more particles were CD9 positive than CD63. Of note, due to the low particle count, the particle to noise ratio (IgG spot) was comparatively low. TP colocalization changed with an increase of dual positive for CD9/CD63 on the CD63 capture spot and increase of single positive on the CD81 spot. On the CD9 capture spot, both CD9 single positive and CD9/CD81 double positive particles increased. Relative IM positive particles decreased again to load level.

Overall, the constant TP composition and colocalization in the EV fractions (elution 5 and 9) indicate that no bias occurs during AEX isolation. The increase in IM-positive particles from elution 5 to 9 indicates that larger EVs elute later and that very few TP-positive EVs were present at the end of the salt gradient confirming the CD81 ELISA data (Figure 3C).

### 3.5 Co-eluting protein contaminations vary in amount and size during elution

**FIGURE 6:**
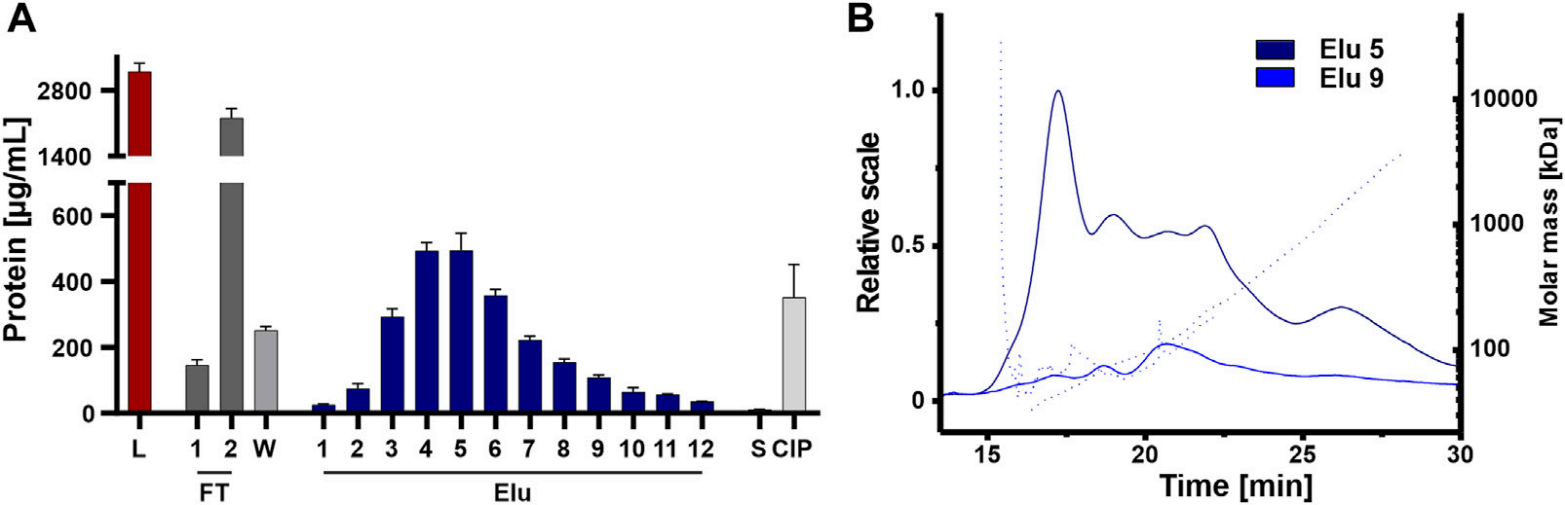
Purity of isolated HEK293 EV. **(A)** Protein concentration of load (L) and chromatographic fractions flow-through (FT), wash (W), elution (Elu), strip (S) and cleaning in place (CIP), measured by bicinchoninic acid assay (BCA). **(B)** Asymmetric-flow field-flow fractionation (AF4) chromatograms of elution fractions Elu 5 (dark blue) and Elu 9 (middle blue). UV 280 traces (continuous line) with distribution of molar masses (dotted lines) are shown.

The elution fractions were further analyzed for coeluting proteins that affect the purity of the isolated EVs, an important factor for therapeutic applications (Bracewell et al., 2015; Pilely et al., 2022). Protein content was analyzed by BCA assay on all steps, including the unpurified load, to assess the purity of the chromatographic steps (Figure 6A). The unpurified EVs had the highest protein concentration, followed by the flow-through fraction 2. The protein concentration decreased during washing and then gradually increased during elution, peaking in elution 4 and 5 with one-sixth of the protein concentration of the load. The late elution fractions and the strip showed minimal protein concentrations, while the CIP showed higher protein concentration with one-seventh the protein concentration compared to the load, which in turn contained more protein. Protein contamination in elution fractions 5 and 9 was also examined in more detail using AF4 analysis (Figure 6B). Elution 5 showed a consistently higher protein content across all protein sizes with small proteins with molecular masses between 45 and 150 kDa and larger proteins with molecular masses >1,000 kDa. Elution 9 showed a low protein content with molecular masses between 50 and 100 kDa.

Even though protein concentration is decreased in the early elution fractions compared the load, proteins appeared to co-elute primarily early during the NaCl gradient and some proteins remain on column and tubing until CIP.

In accordance to the MISEV guidelines (Théry et al., 2018) samples were additionally examined by TEM and Western blot to characterize, as well as visualize EVs and potential impurities. TEM images of the load (Supplementary Figure S2B) showed cup-shaped EVs, partially embedded larger aggregated structures, and small protein aggregates. Elution 5 images also showed cup-shaped EVs with smaller protein aggregates and a less contaminated background. Elution 9 and 11 both showed larger EVs and fewer protein aggregates, with Elution 9 showing more EVs overall.

Western blot analysis of all chromatographic fractions (Supplementary Figure S2A) showed that the EV markers TPs and syntenin-1 were detectable in the load and the elution with the strongest signal starting at elution 2 and declining after elution 10. They were not detectable in the other fractions. Calnexin (CNX), which is used as a marker for Endoplasmic reticulum-derived vesicular contamination (Théry et al., 2018; Wang et al., 2020), could only be detected in the cell lysate. Samples were not concentrated prior WB analysis to reflect protein concentrations and not introduce a bias.

Interestingly, the later elution fractions in the TEM images show less protein contamination and larger EVs.

### 3.6 Nuclease treatment reduces co-isolation of nucleic acids and associated proteins

**FIGURE 7:**
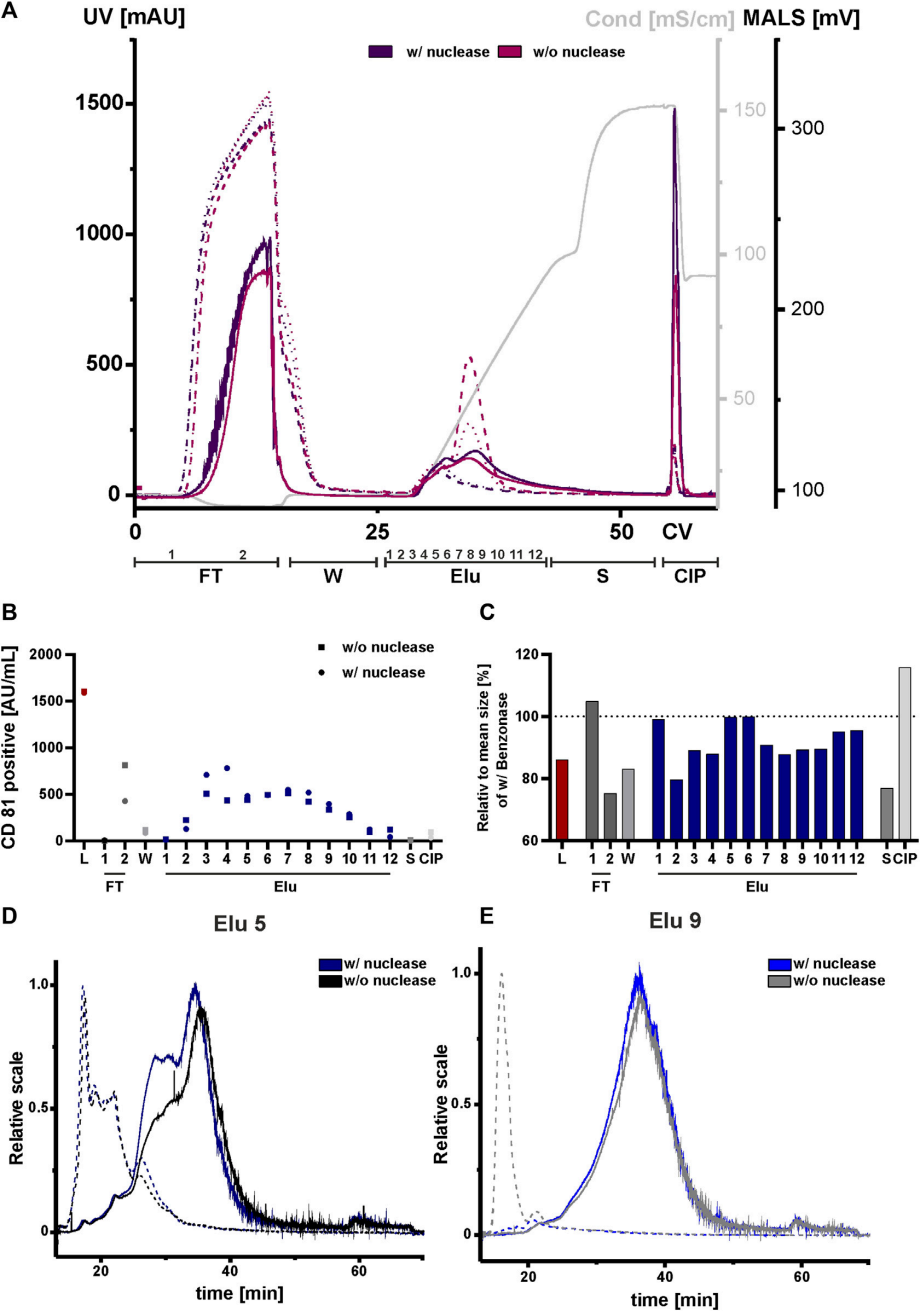
Effect of nuclease digestion on HEK293 EV purification by anion exchange chromatography. **(A)** Overlay of Chromatograms of two representative runs with nuclease pretreatment (run 1, dark purple) and without nuclease pretreatment (run 4, pink) in column volume (CV) with chromatographic steps flow-through (FT), wash (W), elution (Elu), strip (S) and cleaning in place (CIP) and including fraction numbers. UV 260 (dashed line), UV 280 (dotted line) and multiangle light scattering (MALS) (continuous line) traces, as well as conductivity (light grey) are shown. **(B)** Analysis of chromatographic fractions based on the presence of cluster of differentiation (CD)81 by CD81 enzyme-linked immunosorbent assay (ELISA), two technical replicates. **(C)** Distribution of mean size in all fractions, determined by nanoparticle tracking analysis (NTA), five technical replicates each. **(D, E)** Asymmetric-flow field-flow fractionation (AF4) chromatograms of elution fractions Elu 5 and Elu 9, with nuclease pretreatment (run 1, dark blue and middle blue) and without nuclease pretreatment (run 4, black and grey), UV 280 (dashed line) and MALS (continuous line) traces are shown.

Due to their negatively charge, both, nucleic acids and chromatin can compete with EVs and proteins for resin binding sites. This can affect the separation and ultimately the purity of the elution. We therefore tested nuclease pretreatment prior to the concentration step to analyze the influence and possible interference of negatively charged nucleic acids and chromatin, DNA with DNA-binding proteins on the chromatography run. All fractions were again analyzed by NTA, ELISA, AF4 and Western blot. An overview of the conducted runs with and without nuclease pretreatment is shown in the chromatogram in Figure 7A. The elution profile without nuclease digestion prior to the chromatography run showed a second peak with a peak maximum of 520 mAU and 250 mAU in the UV trace at 260 and 280 nm at 33 CV. The UV traces in the flow-through, strip and CIP peaks were comparable to those in the nuclease pretreated run. Throughout all chromatographic steps, differences in the peak maximum measured by MALS were observed. The peak maxima were consistently lower in the undigested run when compared to the run 1 with the addition of the nuclease treatment. In addition, the MALS signal revealed (Figure 7A), that the resolution of the two peaks without nuclease digestion was lower. Taken together, these data indicate the presence of both DNA and chromatin in the second UV peak revealed by the reduced MALS signal in the treated chromatography run.

We then performed an ELISA for specific quantification of CD81 on the vesicles to analyze the chromatographic fractions with and without nuclease treatment (Figure 7B). The results of the ELISA showed almost twice as many CD81-positive EVs were present in flow-through 2 without the nuclease digestion. Additionally, approximately 15% fewer CD81-positive EVs were eluted compared to the samples with prior nuclease digestion. For both conditions the signals of CD81-positive EVs in the other fractions, including load, remained unchanged.

NTA analysis revealed differences in particle size (Figure 7C). The particle size of the undigested fractions was smaller than of the nuclease-treated fractions, where a particle size reduction ranging between 10 and 40 nm was measured. Again, AF4 was utilized for an in-depth analysis of the elution fractions (Figures 7D,E). In elution fraction 5, which is equal to peak 1 from the chromatography run shown in Figure 2 (Figure 2), the MALS signal of the two sample treatments differed at 30 min. However, in the UV traces the signals were almost identical. The situation was different for elution fraction 9, which corresponds to the second peak of the chromatographic separation (Figure 2). Here, the MALS signals were comparable. In the UV signal of the run without nuclease digestion, a distinct UV peak was observed at 10 min, indicating the presence of nucleic acids/chromatin due to its absence in the nuclease-treated sample. By contrast, elution fraction 9 of the nuclease treated run had no UV signal at 10 min (Figure 7E). This was further supported by Western blot analysis (Supplementary Figure S3B). Staining of the nucleic acid-associated protein histone 3 showed a slight decrease in the elution fractions of the digested sample, whereby the CD81 protein level was not affected. Interestingly, the size distribution for peak 1 and 2 in the AF4 MALS measurements showed smaller particles in the chromatography run with nuclease digestion when compared to the run without pre-treatment (Supplementary Figure S3A). This observation was contrary to the results obtained by NTA (Figure 7C).

In conclusion, treatment of the load with nuclease revealed differences in impurities, particle sizes and number of eluted CD81-positive EVs in the chromatographic runs when compared to non-treated load. Discrepancies in the NTA and AF4 measurements could be explained by the limited sensitivity of the NTA for particles with sizes <50 nm (Vogel et al., 2021). However, AF4 measurement indicated that these particles represented the largest population of particles.

### 3.7 AEX method is applicable to EVs from other cell sources and gives comparable results

EVs were also isolated from Jurkat cells to ensure that the method for isolating EVs with Eshmuno^®^ Q from cell culture of different cell types was applicable. The same volume of CM was prepared in an identical manner to that of the HEK293 cells and analyzed using a variety of methods (Supplementary Figure S5–S7). The load from Jurkat cells (Sup Figure 5B) showed a much lower concentration of particles, resulting in no particle breakthrough and few particles in FT and wash fractions (Sup Figure 5). The elution profile of Jurkat EVs was similar to HEK293 EVs with 2 peaks during elution, detectable by MALS and NTA (Sup Figure 5A+B), with the difference that peak 1 starts earlier and is higher compared to peak 2. UV adsorption chromatogram and CD81 ELISA (Sup Figure 5A+D) showed only one peak at the beginning of the elution, like HEK293 EVs, Jurkat EVs revealed a different TP profile than HEK293 EVs with CD81 followed by CD9 but the composition remained similar throughout the elution, with fewer particles present in elution fractions 9 and 11. Protein elution profile remained similar to HEK293 EVs (Sup Figure 7A) with protein contamination and 2 particle peaks detectable in elution fraction 3, but only small amounts of protein and one particle peak in elution fraction 9 (Sup Figure 7C). The mean particle size showed a similar profile with the exception that Elu 3 had an increased particle size (Sup Figure 7B).

## 4 Discussion

Scalability is a drawback of current EV isolation techniques such as ultracentrifugation or size-exclusion chromatography (Burgess, 2018; Hall, 2018; Lee et al., 2020). Initial work has been published, describing IEX as a potential method for EV isolation and purification (Kim et al., 2016; Kosanović et al., 2017; Heath et al., 2018; Silva et al., 2023). In this study, we demonstrate that AEX chromatography combined with the tentacle technology is a suitable and scalable method for isolating and purifying EV. High product recovery of more than 70% was achieved, while we revealed that a significant amount of EVs were lost in the chromatography system due to adsorption.

Understanding the chromatographic process through in-depth analysis of all fractions allowed us to determine the binding and elution behavior of EVs to the chromatographic material, as well as the possible presence of impurities such as host cell proteins (HCP) and host cell DNA (Rathore et al., 2003; Kornecki et al., 2017). The advantages and limitations of each analytical technique were also discussed in this study. The analysis of EVs is complex and challenging. Therefore, we have implemented a variety of analytical methods to best monitor and understand the entire process. Here we used in-line MALS, NTA and AF4-UV-MALS for particle concentration and size characterization. Inline UV detection, BCA analysis, AF4-UV-MALS, Western blot and TEM were used for purity determination. ExoView™ R100 platform, Western blot and ELISA were used for precise characterization of specific surface markers and their colocalization. The robustness of the data obtained is improved by implementing several methods of analysis in parallel (Shao et al., 2018; Kwon and Park, 2022; Davidson et al., 2023). Particularly important for the comparability of the data is the specification of the exact measurement parameters, which is also implemented in the MISEV 2018 guideline (Théry et al., 2018).

The variance in measurement results we observed, increased as the sample heterogeneity increases, such as loading, throughput, and CIP. Since NTA determines recovery based on particle concentrations, there is some variance, especially in determining the load to which the recovery relates (Vestad et al., 2017; Vogel et al., 2021). Optical methods such as NTA are only capable of determining paricles semi-quantitative. Smaller particles with a lower refractive index are often missed due to the lower resolution (Sitar et al., 2015; Hendrix et al., 2023). Further, it cannot distinguish between membrane-bound EVs, lipids and protein aggregates. Notably, fluorescence-based NTA is emerging as a new method for counting and phenotyping EVs that have previously been fluorescently labeled with non-specific membrane markers or with antibodies that specifically recognize EV surface marker proteins. However, this method was not available for this study (Desgeorges et al., 2020).

In particular, MALS detection is subject to a large degree of variation when only a small number of large particles are present. Since the number of particles is calculated from the size distribution, it is necessary to check the data for consistency (Sitar et al., 2015; Shao et al., 2018). Despite this drawback, the method is well suited for inline particle monitoring in chromatography. Combining MALS with AF4 is also recommended here, as the first step involves separating according to size. This reduces the influence of large particles on the overall result. In addition, AF4-MALS allows separation and detection of subpopulations, whereas NTA can only determine a broad size distribution. Differentiation between particles and EV is not possible with any of the particle methods. Therefore, a method such as ELISA, ExoView™ R100 platform or Western blot is mandatory for detection of EV based on EV-related proteins or surface proteins. Samples were diluted to a narrow concentration window prior to measurement to reduce variation in concentration determination with NTA. The application of these methods to the samples showed that both particles and CD81-positive EVs could be detected in the flow-through. This is an indication that we have been working above the binding capacity of the column. When the particle concentration was halved, there was still no breakthrough, indicating that the maximum binding capacity is more than 7.5 × 10^10^ particles/mL resin. The particle concentration in the eluate was comparable to others (McNamara and Dittmer, 2020).

The NaCl gradient in the elution partially separated two subpopulations that differed in particle size. CD81 positive EVs were detectable in the elution, but their abundance decreased towards the end of the elution. Fractions eluting at lower salt concentrations were likely dominated by EVs characterized by CD81, CD63 and low levels of CD9 on the EV surface. In this study, we were able to show for the first time the colocalization of TP during a chromatographic run. Interestingly, the colocalization and composition of the three TPs does not appear to change during elution. This suggests that no bias toward a specific TP-positive population is induced during chromatographic isolation. Changes in TP pattern of the EV populations in the late elution fraction are most likely due to a lower overall particle concentration and an increase in interfering conditions such as high NaCl concentration. Particles were also detected in the strip and CIP fractions, while the CD81 ELISA and Western blot did not show signals for EV-characteristic proteins. We suggest that EVs still bound to the column or tube, including EV-associated protein, would most likely be degraded during the harsh CIP conditions and would not be detected (Wiest et al., 1988; Cheng et al., 2019).

Overall, this observation indicates that TP-positive EVs were eluted earlier than other vesicles such as TP-negative EVs. Possibly, these particles were more strongly bound to the stationary phase and released later. This is in line with the increase in particle size during elution. It is possible that this is due to the larger surface area and thus higher negative charge or a change in the glycan pattern (Akagi et al., 2014). The tentacle structure of the chosen resin would then be responsible for more efficient binding to the surface of the EV, thus increasing the interaction strength. Our results are also in agreement with the studies of Kosanovic et al. and Seo et al. (Kosanović et al., 2017; Seo et al., 2022).

AEX resins are widely used in a variety of bioprocesses (Wallace and Rochfort, 2023), including the purification of monoclonal antibodies and nucleic acids (Eon-Duval and Burke, 2004). By choosing the right process conditions, AEX materials can bind preferentially to one specific target present in the load over another due to different target charges. At the same time, all process-related impurities, such as HCP and DNA, and product-related impurities, such as aggregates, must be removed from the final product or must not exceed a certain level. The limits are set by the Food and Drug Administration (FDA) or the European Medicines Agency (EMA) for biological therapeutic products (Moleirinho et al., 2020). Our data show that EV and proteins were present in the elution fractions. Our observation suggests that proteins may coelute or associate with EVs, possibly by charge or protein-protein interaction. This result is consistent with the data shown by Seo et al. (Seo et al., 2022).

In addition to HCPs, our various analytical methods revealed the presence of nucleic acids. The comparison between nuclease-treated and untreated loading material showed differences in particle size and number of eluted CD81 positive EVs. The size differences indicate that the EVs were associated with nucleic acids (Shelke et al., 2016; Németh et al., 2017), as digestion of these surface-bound nucleic acids results in an overall reduction in particle size. As far as we know, the influence of this removal of EV-surface associated nucleic acids on the functionality of the EV has not yet been conclusively clarified. In addition, the use of nuclease as a pre-treatment may be advantageous, since our data not only showed more CD81-positive EVs in the flow-through in the untreated chromatography run, but also the digestion of nucleic acids can reduce the viscosity of the load, thus lowering the shear stress (Busatto et al., 2018).

As can be seen from the analysis results of the AF4 with pre-treated feed, ultrafiltration results in the formation of aggregates. These larger aggregates of vesicles interact less with the column material than EV and are in the flow-through. Therefore, no effect on the chromatography run was observed. Nevertheless, aggregation of EV reduces yield and recovery and should be avoided. Since the concentration and the associated ultrafiltration step (Arakawa et al., 2017) are suspected to promote this aggregation, the use of a membrane instead of the column with similar/same surface modification could help. Due to the higher flow rates and thus faster processing of the feed, a concentration step can be omitted here. This not only reduces variability, but also costs, process time and productivity.

The knowledge gained about the elution behavior of the product and the process-related impurities present in the feed can now be used to determine further pretreatment steps of the feed material prior to chromatographic separation. In addition, the process knowledge can now be used to optimize the method by selecting chromatographic conditions capable of separating the product from the impurities. Chromatographic purification can be positively influenced by appropriate feed pretreatment methods. Nucleic acids, DNA-binding proteins and proteins in general can compete with the vesicles for binding sites (Kramberger et al., 2015; Arakawa et al., 2017; Beck et al., 2023). Therefore, appropriate pre-purification such as nuclease pretreatment or ultrafiltration and TFF can increase EV binding capacity and further reduce contaminants (Corso et al., 2017; Staubach et al., 2021). Adjusting the conductivity of the feed to reduce or prevent coelution with proteins may be another way to reduce competition for binding sites (Silva et al., 2023). Although it may be possible to separate viruses from EVs by AEX by adjusting the elution conditions (gradient and buffer conditions), this will most likely have a negative impact on production time and yield. Therefore, it is preferable to ensure that no viral contamination is introduced during the EV production and isolation process, which is mandatory in a pharmaceutical setting (Barone et al., 2020). Besides HEK293 cells as a pharmaceutical standard cell line, EVs from mesenchymal stem cells (MSCs) might also be of interest for large scale isolation. EVs from MSCs are currently investigated in clinical trials as therapeutics for their regenerative properties (Gowen et al., 2020). With the results from HEK293 and Jurkat EVs, it is plausible that also EVs from other cellular sources are isolatable by AEX and follow a similar elution profile. Depending on cell type and EV surface charge, elution conditions could be optimized to the individual cell line.

This study demonstrated for the first time that 20%–30% of EVs remain attached to the tubes and chromatography system during EV purification. Further studies should address the absorptivity and overall stability of the EVs. In particular, the addition of additives already used in the pharmaceutical industry for the formulation of other biomolecules could be a solution to limit the effect of adsorption or to increase stability. For example, trehalose has already been described as a possible additive in EV preparations for stabilization (Jeyaram and Jay, 2017; Görgens et al., 2022) and avoidance of aggregation (Jain and Roy, 2009). This could be used as a starting point for an appropriate study.

Overall, our study shows that in the development of future EV isolation techniques, the analytical parameters as well as the recovery rate and other related parameters should be specified in detail. This will allow better comparability and contribute to the development of scalable EV isolation techniques. The results of the analysis of the individual fractions and their impurities can be used to optimize the chromatographic method.

## Data Availability

The original contributions presented in the study are included in the article/Supplementary Material, further inquiries can be directed to the corresponding author/s.
