# Supplementary material for: Novel insights into the isolation of extracellular vesicles by anion exchange chromatography
Source: Front Bioeng Biotechnol. 2024 Jan 19;11:1298892. doi: 10.3389/fbioe.2023.1298892 (PMC10836363; doi:10.3389/fbioe.2023.1298892)
Supplement: Supplementary file 1 [file DataSheet1.docx]

Supplementary Material

Novel insights into the isolation of extracellular vesicles by anion exchange chromatograph

Leon F Koch^1†^, Tatjana Best^1,2†^, Elena Wüstenhagen^2^, Klaus Adrian ^2^, Oliver Rammo^2^, Meike J Saul^3,1^*

^1^Department of Biology, Technische Universität Darmstadt, 64287 Darmstadt, Germany

^2^Merck Life Science KGaA, 64293 Darmstadt, Germany

^3^ Department of Oncology, Hematology and Bone Marrow Transplantation with Section Pneumology, University Cancer Center Hamburg, University Clinic Hamburg-Eppendorf, 20251 Hamburg, Germany

**† These authors contributed equally to this work and share first authorship**

*** Correspondence:**Meike J Saul
[M.Saul@uke.de](mailto:M.Saul@uke.de)

Supplementary table 1. Composition and pH of buffers used for EV isolation. Buffers were adjusted with 2M HCl and filtered through 0.1 µm membrane filter.

|  | Wash buffer | Buffer B |
| --- | --- | --- |
| NaCl [mM] | 125 | 2000 |
| TRIS [mM] | 50 | 50 |
| pH | 7.4 | 7.4 |

Supplementary table 2. Detailed information on all individual chromatography steps.

| Step | CV | Flow rate [ml/min] | Solution |
| --- | --- | --- | --- |
| Equilibration | 10 | 1 | Wash Buffer |
| Sample Application | N/A | 0.5 | Conc. CM |
| Wash | 10 | 1 | Wash Buffer |
| Elution | 15 | 1 | 0-60 % Buffer B |
| Strip | 10 | 1 | 100% Buffer B |
| Cleanin in Place (CIP) | 10 | 1 | 0.5 M NaOH |
| Re-equilibration | 10 | 1 | Wash Buffer |


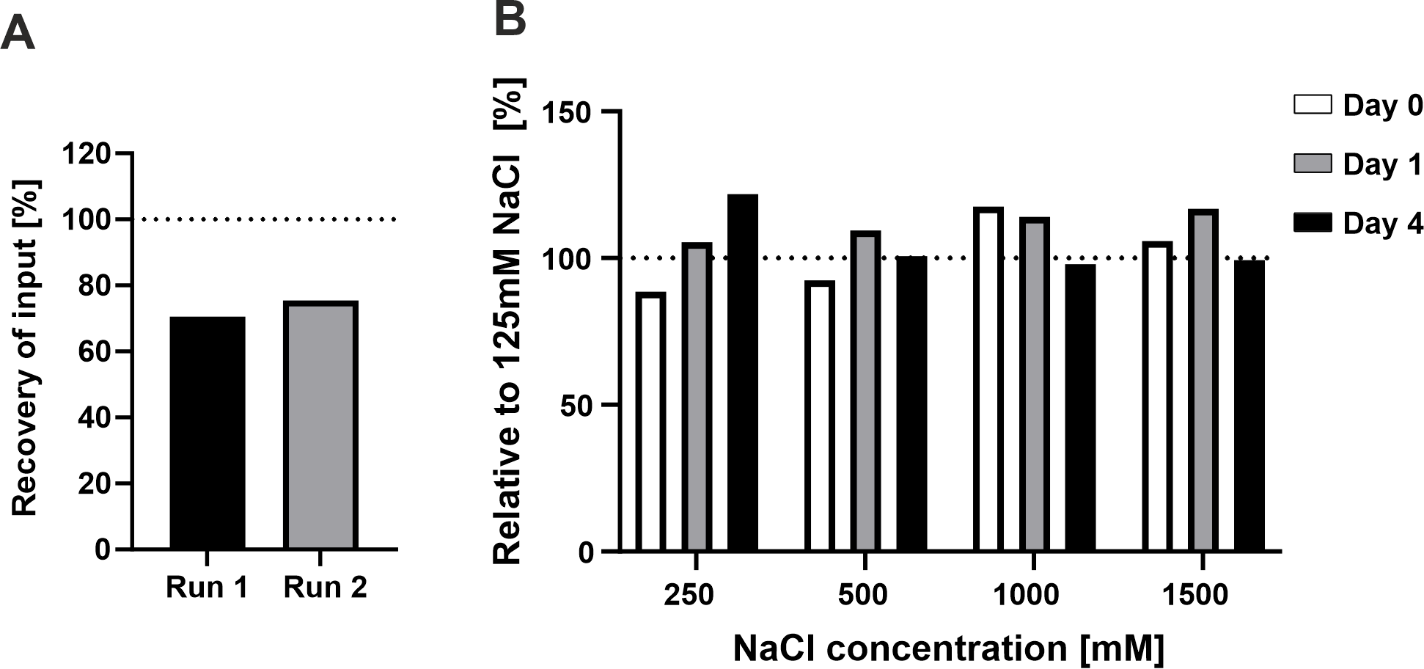


Supplementary Figure 1. A) Recovery of isolated EVs after applied to chromatography system without column. B) Stability of isolated EVs at different NaCl concentrations over the course of 4 days at 4°C.


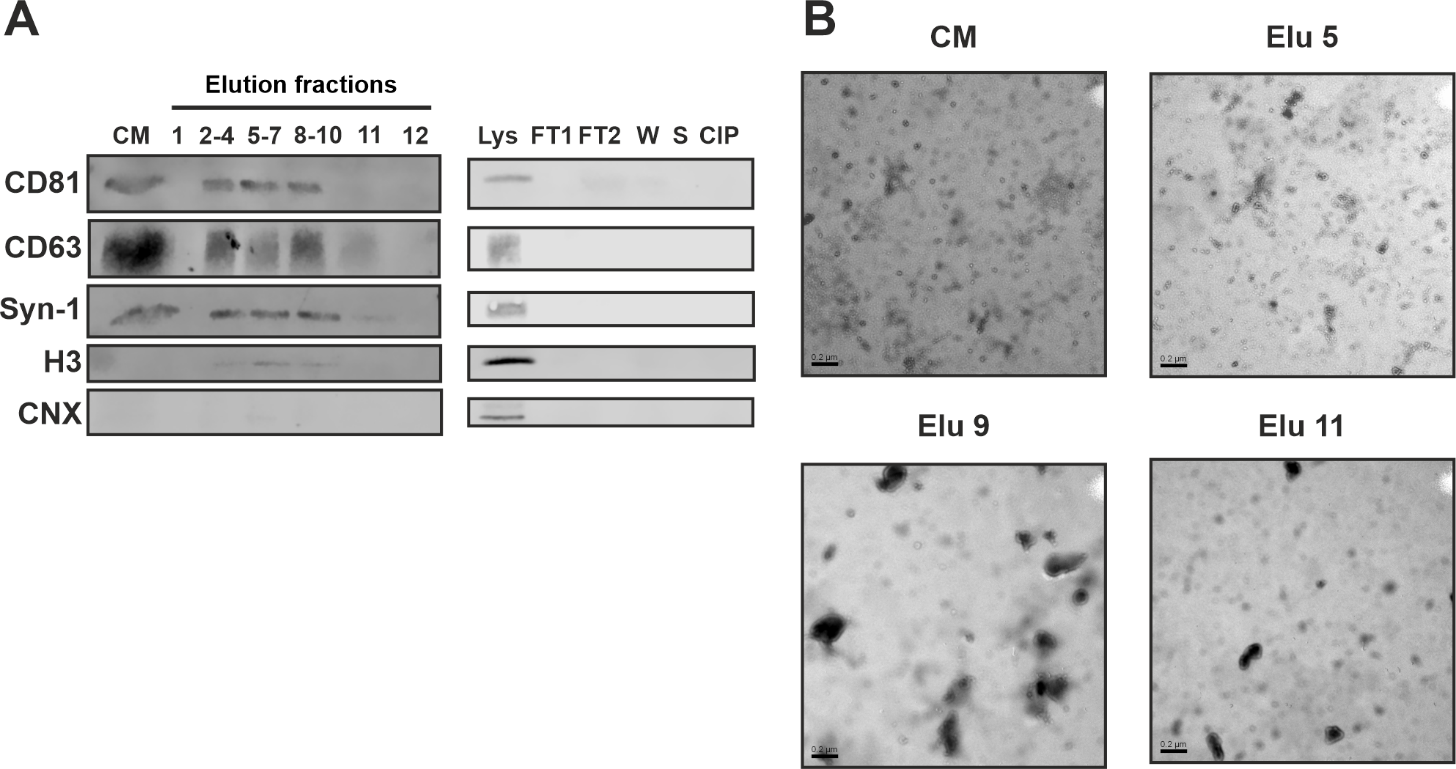


Supplementary Figure 2.A) Western Blot analysis of representative run 1 with chromatographic steps: flow-through (FT), wash (W), elution (Elu), strip (S) and cleaning in place (CIP) as well as Load and HEK293 cell lysate. Depicted are the tetraspanins CD63 and CD81, as well as Syntenin-1 (Syn-1), Histone H3 and Calnexin (CNX). B) Transmission electron microscopy images of conditioned media, Fractions Elu 5,9 and 11. Scalebar represents 200 nm.


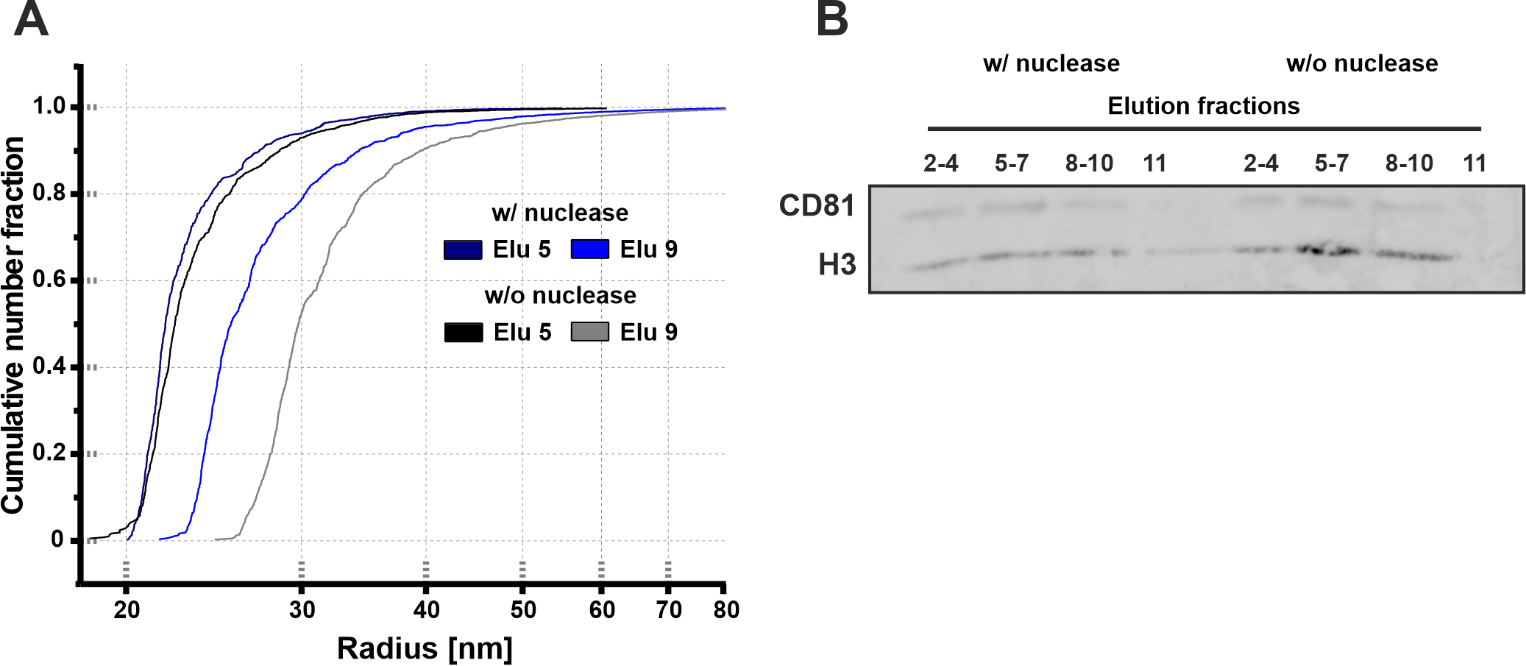


Supplementary Figure 3. A) Cumulative particle sizes of Elution fractions 5 and 9 with and without nuclease treatment. B) Western Blot analysis of elution fractions from chromatography runs with and without nuclease treatment. Depicted are the tetraspanin CD81 and the Histone H3 protein.


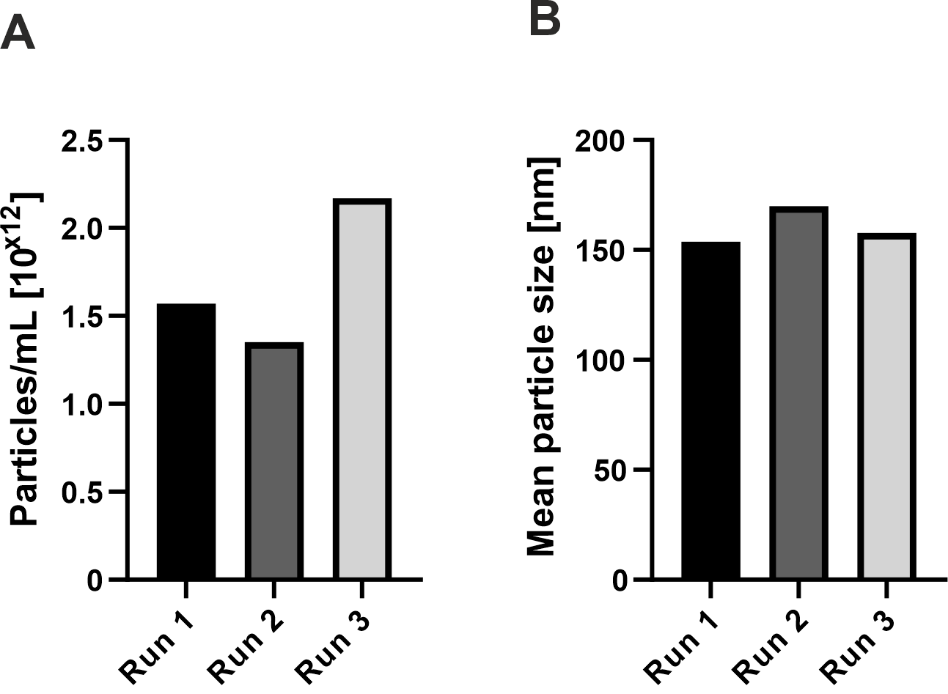


Supplementary Figure 4. Particle concentration (A) determined by nanoparticle tracking analysis and mean particle size (B) of the three concentrated HEK293 conditioned media used in chromatographic isolation.


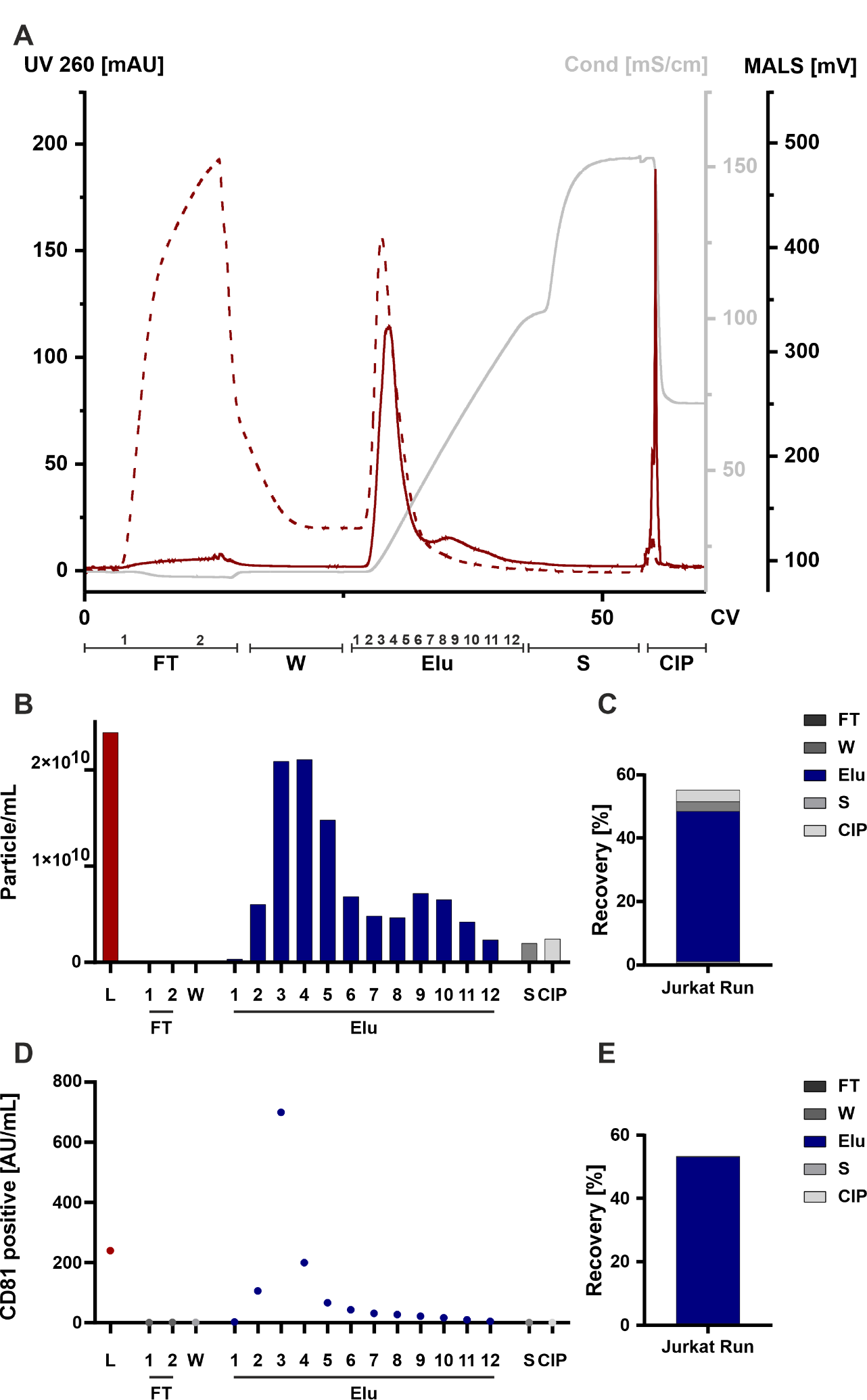


Supplementary Figure 5. EVs from Jurkat cells can bind to and be eluted from a strong anion exchange resin. A) chromatogram of Jurkat concentrated conditioned medium, in column volumes (CV) with chromatographic steps flow-through (FT), wash (W), elution (Elu), strip (S) and cleaning in place (CIP) and including fraction numbers. UV 260 (dashed line) and multiangle light scattering (MALS) traces (continuous line), as well as conductivity (light grey) are shown.


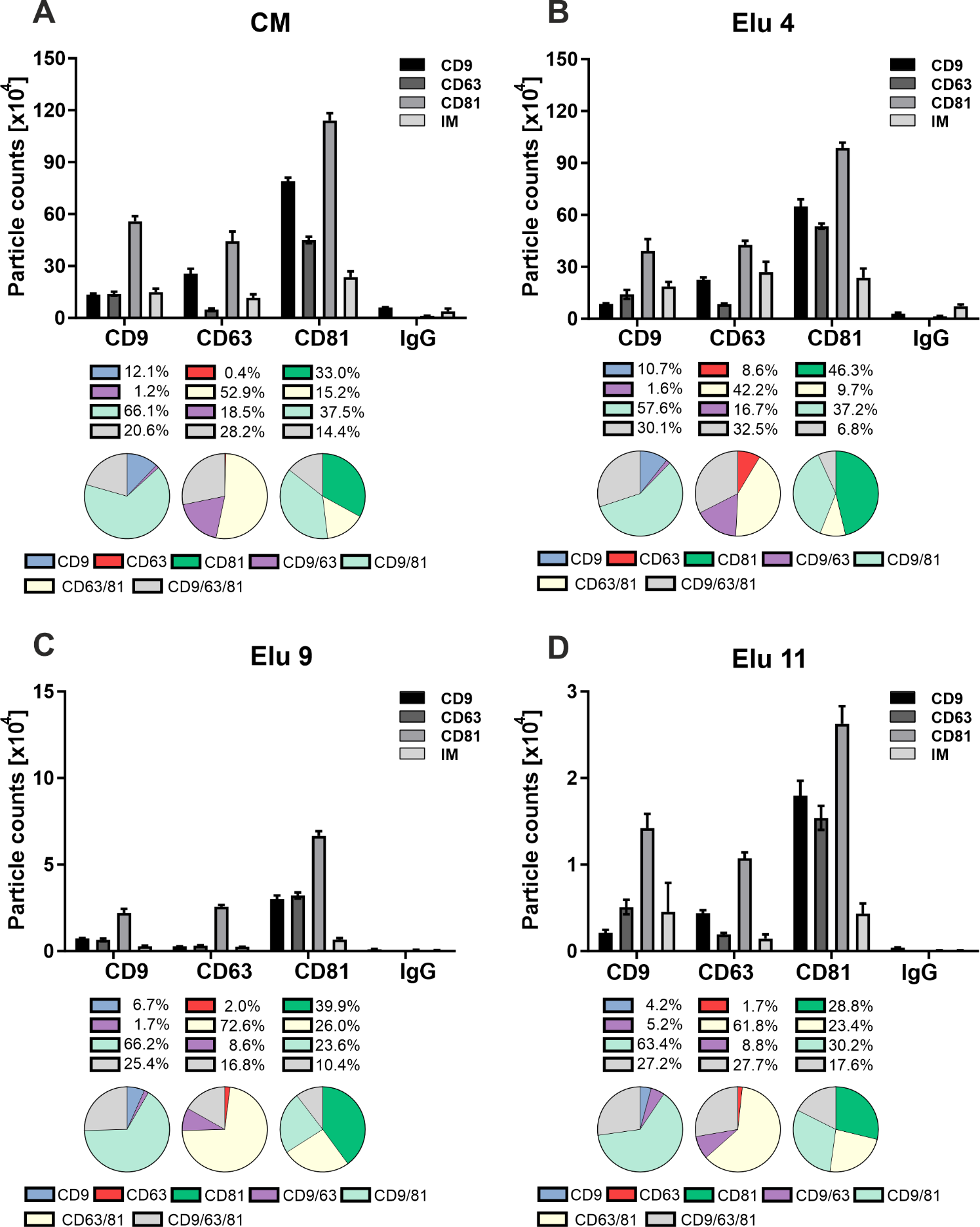


Supplementary Figure 6. Comparison of tetraspanin (TP) composition and colocalization of unpurified EVs from Jurkat cells and elution fractions throughout the NaCl gradient. EVs were captured using chips coated with spots against CD9, CD63, CD81 and mouse IgG as control. Fluorescent antibodies against the same TP were used for visualization and analyzed on the ExoView™ R100. A) depicts TP composition and colocalization of EVs present in the load. B-D) display the composition and colocalization of the elution fractions Elu 4, Elu 9 and Elu 11, respectively. Interferometric positive particles were detected using the SP-IRIS mode, with a detection threshold of 50 nm.


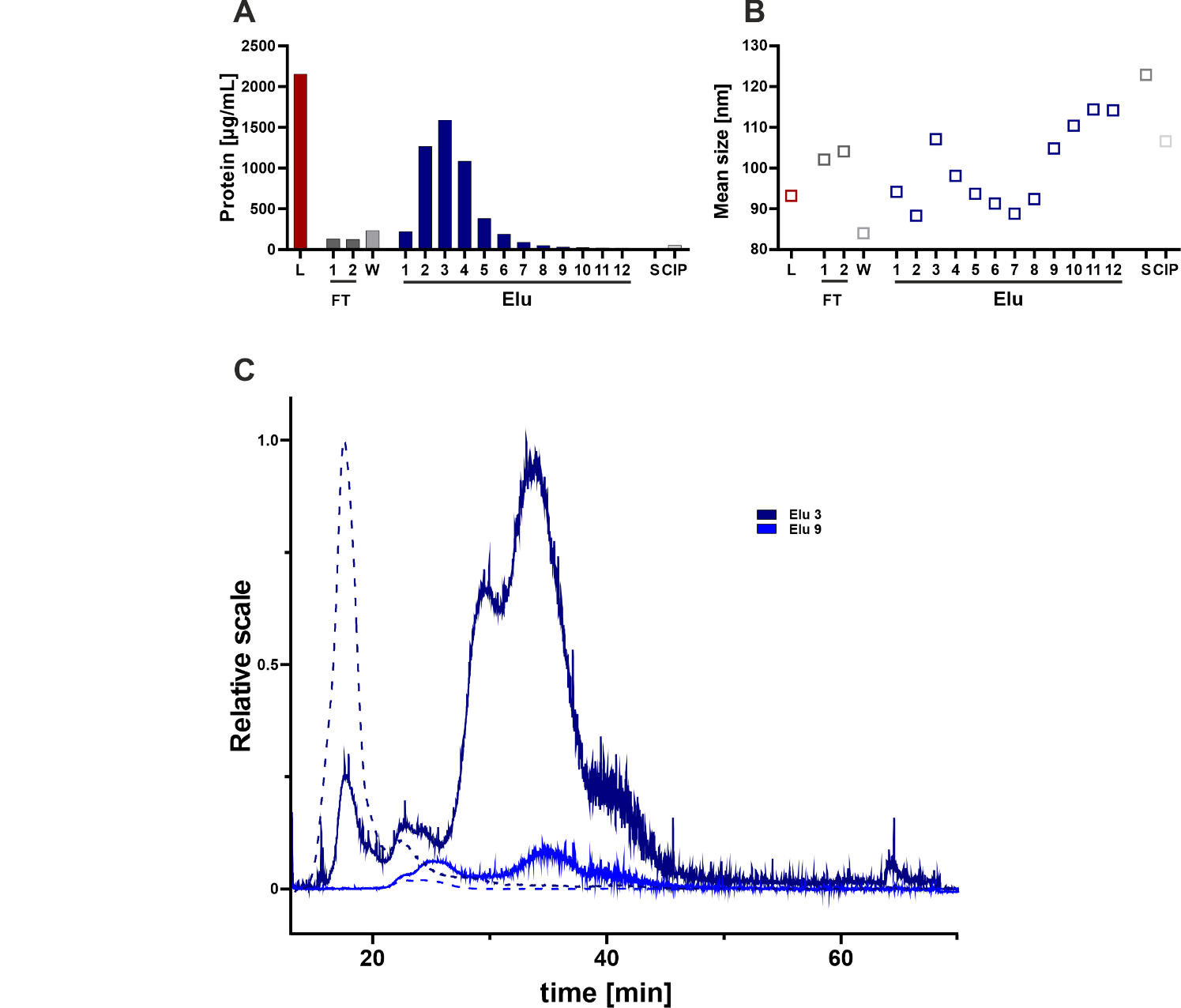


Supplementary Figure 7. Purity of isolated Jurkat EV. A) Protein concentration of load (L) and chromatographic fractions flow-through (FT), wash (W), elution (Elu), strip (S) and cleaning in place (CIP), measured by bicinchoninic acid assay (BCA). B) Mean vesicle sizes of L and chromatographic fractions FT, W, Elu, S and CIP, measured by nanoparticle tracking analysis (NTA). C) Asymmetric-flow field-flow fractionation (AF4) chromatograms of Elu 3 (dark blue) and Elu 9 (light blue), UV 280 (dashed line) and multi-angle light scattering (continuous line) traces are shown.
